# Comparison of ProSeal Laryngeal Mask Airway (PLMA) Placement Using Introducer Tool and Stylet in Neutral Head Position in Adult Patients Under General Anaesthesia

**DOI:** 10.7759/cureus.94477

**Published:** 2025-10-13

**Authors:** Dasara Nongbet, Namita Arora, Priyanka Dev, Sanju Sharma, Astha Sahu

**Affiliations:** 1 Anesthesiology and Critical Care, North Eastern Indira Gandhi Regional Institute of Health and Medical Sciences, Shillong, IND; 2 Anesthesiology, Atal Bihari Vajpayee Institute of Medical Sciences (ABVIMS) and Dr. Ram Manohar Lohia (RML) Hospital, New Delhi, IND

**Keywords:** airway management, bronchoscope, general anaesthesia, laryngeal masks, stylet

## Abstract

Background: Airway management in patients with cervical spine injuries presents significant challenges during general anaesthesia. The ProSeal laryngeal mask airway (PLMA) has emerged as a viable alternative for difficult airway management. However, optimal insertion techniques in neutral head positioning remain unclear. This study compared PLMA insertion using an endotracheal stylet versus the standard introducer tool for patients requiring neutral head positioning, with placement quality assessed using fibre-optic bronchoscopy.

Methods: A prospective, randomized controlled trial was conducted with 60 patients undergoing elective surgery requiring PLMA insertion in a neutral head position. Patients were randomly allocated to the introducer tool group (Group IT, n=30) or the stylet group (Group ST, n=30). The primary outcome was PLMA placement quality using Brimacombe's fibre-optic scoring system. Secondary outcomes included first-attempt success rate, number of insertion attempts, insertion time and haemodynamic responses. Statistical analysis was performed using chi-square and unpaired t-tests, with p<0.05 considered significant.

Results: Optimal placement (grades 3 and 4) was achieved in all patients in both groups, with no significant difference in Brimacombe scoring between Group IT and Group ST (p=0.76). Group ST demonstrated a significantly shorter insertion time (79.37±18.74 seconds) compared to Group IT (96.40±22.92 seconds, p=0.002). No significant differences were observed in first-attempt success rate, haemodynamic parameters or insertion attempts between groups.

Conclusion: Both techniques achieved excellent PLMA placement quality in neutral head positioning. The stylet technique demonstrated significantly faster insertion times while maintaining comparable success rates and haemodynamic stability. These findings suggest that the stylet technique may offer practical advantages for PLMA insertion in patients requiring cervical spine immobilization, potentially improving efficiency in challenging airway scenarios.

## Introduction

Supraglottic airway devices (SADs) offer a less invasive alternative to endotracheal intubation during general anaesthesia, requiring minimal expertise and avoiding the haemodynamic response associated with laryngoscopy [1]. The ProSeal laryngeal mask airway (PLMA) incorporates a double-cuff design and an integrated drainage tube to enhance airway seal and reduce aspiration risk compared with the classic laryngeal mask airway (LMA) [2]. However, the standard PLMA introducer tool often fails to reach the device's tip, leading to cuff folding and potential insertion trauma. Various adjuncts, including suction catheters, bougies, flexistylets and fibre-optic bronchoscopes, have been employed to guide PLMA placement, yet each carries a risk of mucosal injury if it extends beyond the tip. Myatra et al. reported that a lubricated endotracheal tube stylet, inserted into the drainage channel and shaped along the PLMA curvature, prevents tip folding without protrusion, thereby minimizing trauma [3].

Airway management in patients with suspected cervical spine injury is further complicated by manual in-line stabilization (MILS), which restricts neck movement [4]. Although the intubating LMA (ILMA) is designed for neutral-head insertion, the PLMA's curvature favours a sniffing position and has not been widely studied under neutral alignment [5].

This study compared PLMA insertion using a standard endotracheal tube stylet versus the manufacturer's introducer tool in adult patients under general anaesthesia with neutral head positioning and assessed correct device placement using fibre-optic bronchoscopy. We hypothesized that stylet-guided PLMA placement would be comparable to introducer-guided placement in terms of insertion success and placement quality.

## Materials and methods

The primary objective of this study was to assess PLMA placement quality using Brimacombe's fibre-optic laryngoscopic (FOL) score [6]. The secondary objectives included first-attempt success rate, number of insertion attempts, total insertion time and haemodynamic response to insertion.

This prospective, randomized controlled trial was conducted in the department of anaesthesiology at a medical college in Northern India, from November 2019 to September 2021. Ethics committee approval was obtained (F.No.TP(MD/MS)(17/20219)/IEC/ABVIMS/RMLH 681/19) and the study was registered with Clinical Trials Registry- India (CTRI) (CTRI/2020/09/027523). Written informed consent was obtained from all participants.

The correction that needs to be made is the sample size was calculated using G*Power v3.1 based on prior favourable Brimacombe grades (3 and 4) [3], demonstrating that 30 patients per group would provide 80% power to detect a difference at α=0.05 (two-sided). Sixty patients were randomized 1:1 via a computer-generated sequence. Categorical variables were analysed using chi-square tests and continuous variables using unpaired t-tests. Group allocation was Group IT (n=30) (PLMA insertion using an introducer tool) and Group ST (n=30) (PLMA insertion using a stylet).

Eligible participants were adults aged 18-60 years, with American Society of Anesthesiologists (ASA) physical status I-II, scheduled for elective surgery under general anaesthesia with PLMA. Exclusion criteria were modified Mallampati grade III-IV, thyromental distance < 6 cm, mouth opening < 2.5 cm and body mass index (BMI) ≥ 35 kg/m².

Standard ASA monitors (pulse oximetry, non-invasive blood pressure and electrocardiogram (ECG)) were applied, and baseline readings were recorded. Intravenous access was secured, and patients were preoxygenated for three minutes. Anaesthesia induction consisted of fentanyl 2 μg/kg, propofol 2 mg/kg and vecuronium 0.1 mg/kg, followed by isoflurane or sevoflurane (minimum alveolar concentration (MAC) 1.0 ± 0.2 in O₂:N₂O 50:50). After three minutes of mask ventilation, manual in-line stabilization was applied.

**Figure 1:**
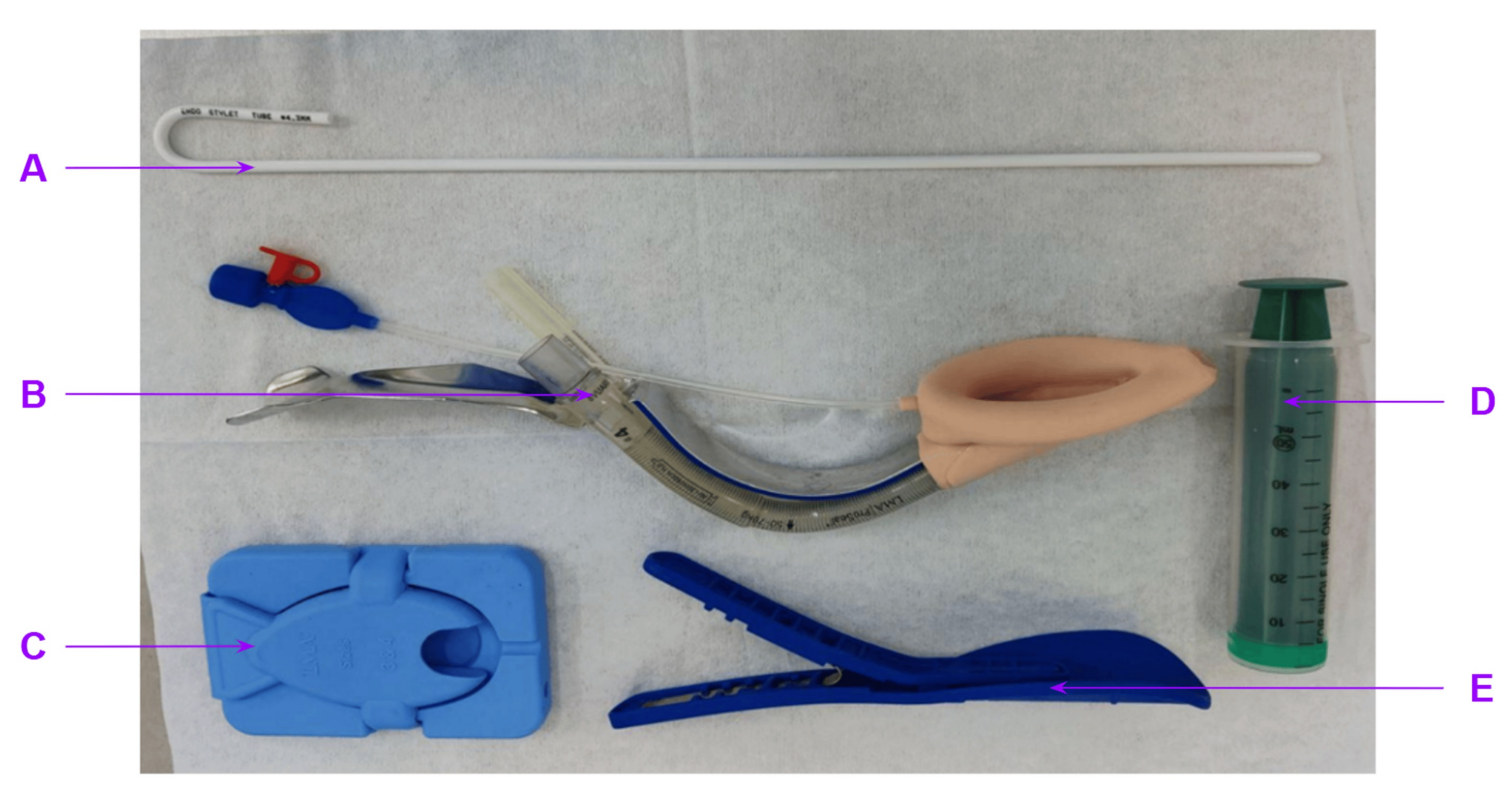
Tools for PLMA insertion A: stylet, B: PLMA with the introducer tool, C and E: types of cuff deflators, D: syringe for cuff inflation PLMA: ProSeal laryngeal mask airway

A size-appropriate PLMA was lubricated, deflated and inserted using the MILS technique. Group IT used the manufacturer's introducer tool; Group ST used the pre-shaped 4.3 mm stylet through the drainage channel to the cuff tip (Figure 1). The stylet was removed post-placement, and the cuff was inflated per the manufacturer's recommendations.

**Figure 2:**
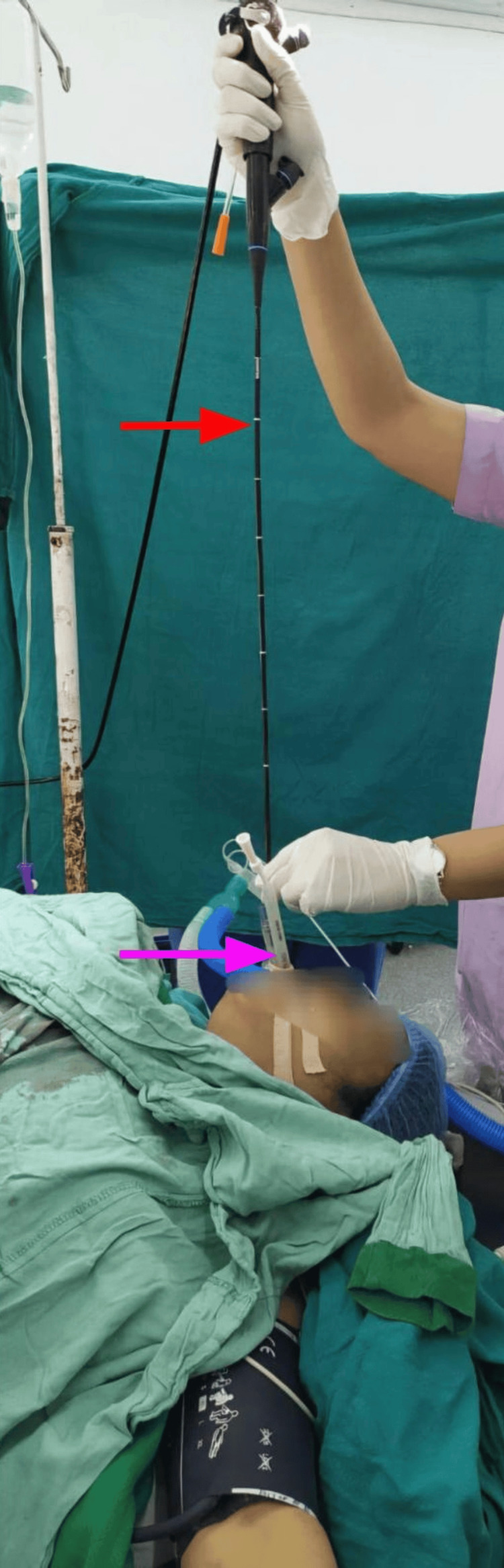
FOB being inserted through the ventilatory port Red arrow: FOB, pink arrow: ventilatory port of the PLMA FOB: flexible bronchoscope, PLMA: ProSeal laryngeal mask airway

The position of the PLMA was checked with a fibre-optic laryngoscopy (Figure 2), and Brimacombe scores of 3 and 4 were considered acceptable (Table 1) [6]. Effective ventilation was defined as bilateral chest rise, peripheral oxygen saturation (SpO₂) ≥ 95%, expired tidal volume ≥ 8 mL/kg and end-tidal carbon dioxide (EtCO₂) < 45 mmHg. Intracuff pressure was maintained at <60 cm H₂O. Haemodynamic parameters (heart rate (HR) and mean arterial pressure (MAP)) were recorded at baseline, immediately post-insertion, every minute for 10 minutes and then every five minutes up to 30 minutes. The time of insertion and number of attempts were documented. Any trauma or blood staining on the devices was noted. A lubricated 14-Fr, 60-cm gastric tube was inserted through the drainage lumen, and placement was confirmed by gastric content aspiration or epigastric auscultation during air injection. A blinded observer performed FOL and clinical assessments. PLMA was repositioned if the oesophageal opening was visible; MILS was removed after confirmation.

**Table 1:** FOL assessment (Brimacombe score) Adapted from Brimacombe J, Berry A. A proposed fiber-optic scoring system to standardize the assessment of laryngeal mask airway position. Anesth Analg. 1993, 76:457-8424538 [6]. Reproduced with permission from Wolters Kluwer Health Inc. and Copyright Clearance Center. FOL: fibre-optic laryngoscopy

Brimacombe score	Fibre-optic view description	Interpretation
0	Failure to function: vocal cords not seen fibre-optically	Unacceptable
1	Vocal cords not seen, but function adequate	Unacceptable
2	Vocal cords plus anterior epiglottis seen	Unacceptable
3	Vocal cords plus posterior epiglottis seen	Acceptable
4	Only vocal cords seen	Acceptable

Anaesthesia was maintained with 2% sevoflurane in 50% O₂/N₂O. Neuromuscular blockade was reversed at the end of surgery, and PLMA was removed once airway reflexes returned. Failed insertions (>3 attempts) were replaced with endotracheal intubation and excluded. Re-insertion attempts, insertion-to-ventilation time and perioperative complications (e.g., dysphagia and sore throat) were recorded.

## Results

**Figure 3:**
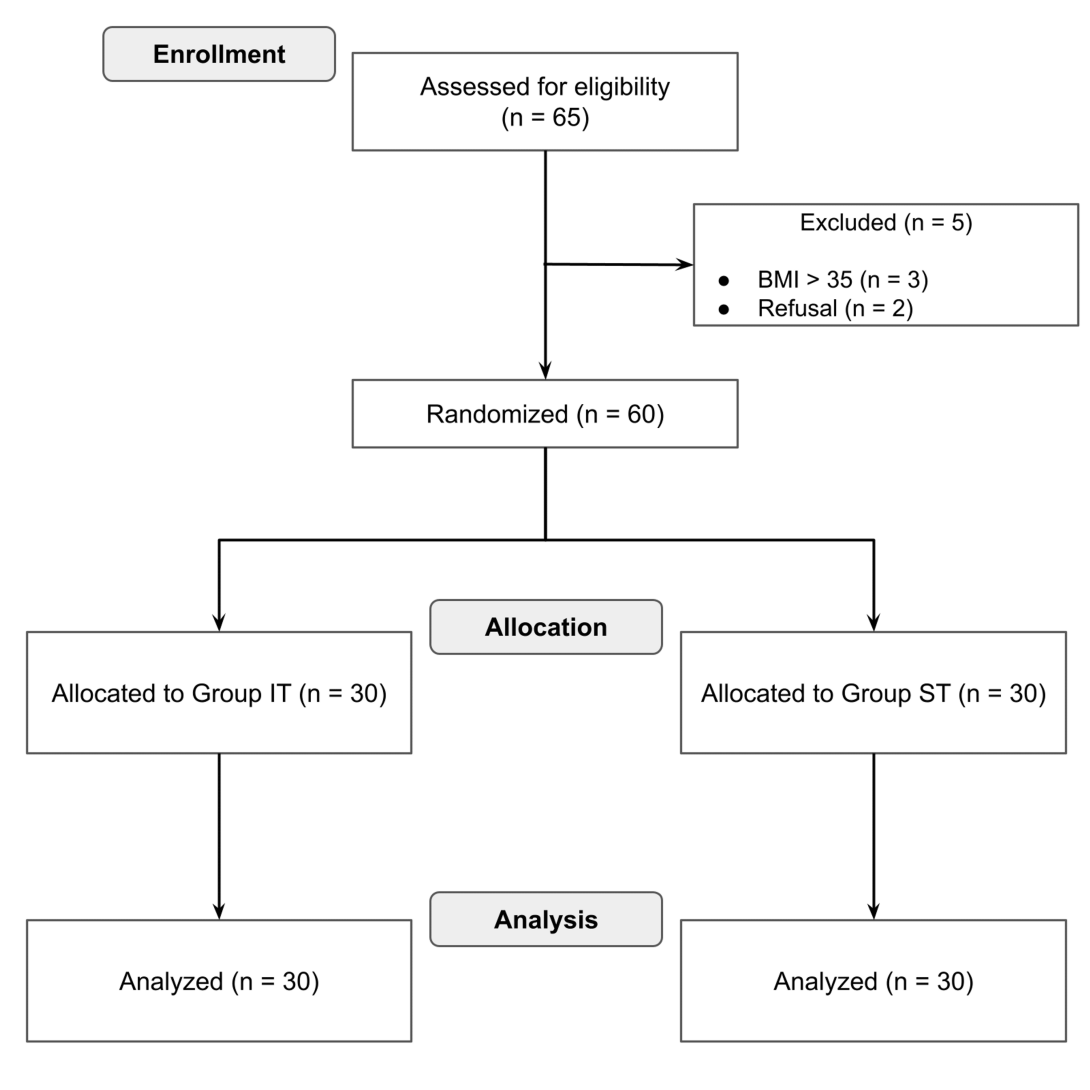
CONSORT diagram showing both Group IT and ST patients BMI: body mass index (kg/cm^2^), IT: introducer tool, ST: stylet, CONSORT: Consolidated Standards of Reporting Trials

A total of 65 patients were screened for eligibility, of whom 60 were included in the study. Five patients were excluded: three due to a body mass index (BMI) ≥ 35 and two who declined participation. Categorical variables were analysed using chi-square tests and continuous variables using unpaired t-tests. The sample size was calculated using G*Power v3.1, demonstrating that 30 patients per group would provide 80% power to detect a difference at α=0.05 (two-sided). The participant flow for the study is summarized in the Consolidated Standards of Reporting Trials (CONSORT) diagram (Figure 3).

This study adhered to predefined inclusion and exclusion criteria, with 60 patients randomized to Group IT and ST. Demographic characteristics, including age and body mass index (BMI), were comparable between groups, as detailed in Table 2. There was no statistically significant difference in gender distribution (p=0.774).

**Table 2:** Demographic data, including age (years) and BMI (kg/m²), of the study subjects The p-value was calculated using an unpaired t-test. IT: introducer tool, ST: stylet, SD: standard deviation, BMI: body mass index

Group		Number	Mean	SD	t-value	p-value
Age (years)	Group IT	30	32.10	12.35	-1.20	0.235
	Group ST	30	36.03	13.03		
BMI (kg/m²)	Group IT	30	21.69	1.15	0.32	0.753
	Group ST	30	21.6	1.05		

Modified Mallampati grade and mouth opening were assessed and found to be comparable between the groups. The PLMA sizes (3, 4 and 5) were selected based on patient weight, with comparable distributions in both groups.

**Figure 4:**
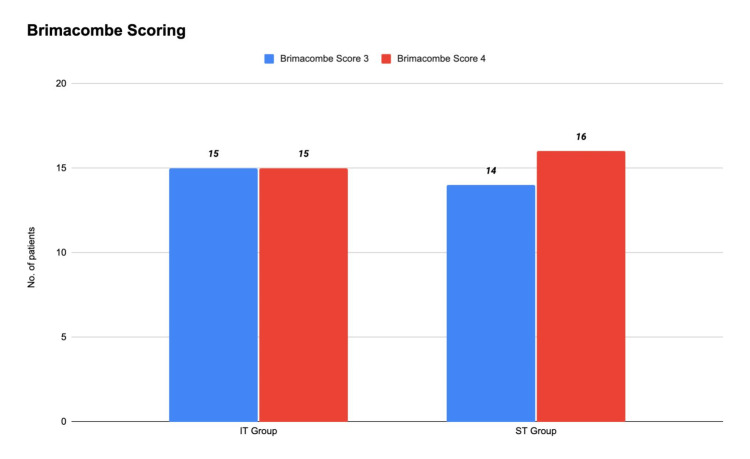
Brimacombe score in the study subjects IT: introducer tool, ST: stylet

The primary outcome (PLMA placement quality assessed by the Brimacombe score) was deemed acceptable at grades 3 and 4. In Group IT, 15 (50%) patients achieved grade 3 and 15 (50%) grade 4, while in Group ST, 14 (46.7%) patients achieved grade 3 and 16 (53.3%) grade 4. These distributions were comparable, indicating that the insertion technique did not significantly affect PLMA positioning (Figure 4).

First-attempt success rates were comparable between Group IT (73.3%) and Group ST (90%) (p=0.095). In Group IT, successful insertions occurred on the first, second and third attempts in 22 (73.3%), 6 (20%) and 2 (6.7%) patients, respectively. In Group ST, corresponding success rates were 27 (90%), 1 (3.3%) and 2 (6.7%) patients. A chi-square test of the distribution of success across attempts (expected counts per group: 24.5 first, 3.5 second and 2.0 third) yielded χ²=2.0 with p=0.130, indicating no statistically significant difference between groups (Table 3).

**Table 3:** Number of attempts in the study subjects IT: introducer tool, ST: stylet

Groups	1st attempt	2nd attempt	3rd attempt	Total
Group IT	22	6	2	30
Group ST	27	1	2	30
Total	49	7	4	60

Group ST had a significantly shorter mean PLMA insertion time (79.37±18.74 seconds) than Group IT (96.40±22.92 seconds), with a p-value of 0.002 (Table 4).

**Table 4:** Time of PLMA insertion in the study subjects An unpaired t-test was used to determine the p-value. IT: introducer tool, ST: stylet, PLMA: ProSeal laryngeal mask airway, SD: standard deviation

Group		Number	Mean	SD	t-value	p-value
Time for insertion (seconds)	Group IT	30	96.400	22.920	3.150	0.002
	Group ST	30	79.370	18.740		

Haemodynamic parameters were recorded at insertion, every minute for 10 minutes and every five minutes thereafter up to 30 minutes. Baseline heart rate (HR 0) was comparable between groups (IT: 80.37±9.21 versus ST: 76.80±9.28; p=0.140). At one minute (HR 1), it was significantly higher in Group IT than in Group ST (90.43±7.28 versus 84.33±9.26; p=0.009). Mean arterial pressure at baseline (MAP 0) and at one minute (MAP 1) did not differ significantly between groups (Table 5). Between-group comparisons were conducted using unpaired t-tests.

**Table 5:** Haemodynamic changes at the time of insertion in the study subjects The p-value was ascertained using an unpaired t-test. IT: introducer tool, ST: stylet, SD: standard deviation, HR: heart rate, MAP: mean arterial pressure

Parameter	Group IT	Group ST	t-value	p-value
HR baseline (mean±SD)	80.37±9.21	76.8±9.28	1.50	0.14
HR at 1 minute (mean±SD)	90.43±7.28	84.33±9.26	2.72	0.009
MAP baseline (mean±SD)	93.33±7.26	94.97±9.33	-0.75	0.46
MAP at 1 minute (mean±SD)	102.26±6.43	99.64±7.44	1.46	0.153

Comorbidities (controlled diabetes mellitus and hypertension) were comparable between groups (p=0.788). Adverse events (blood staining of the PLMA, postoperative sore throat and hypoxia) occurred more frequently in Group IT than in Group ST. No dysphagia or other airway complications were observed.

## Discussion

Supraglottic airway devices (SADs) offer a shorter learning curve compared to endotracheal tubes, making them advantageous for airway management, especially in rescue scenarios. Consequently, SADs are recommended in difficult airway algorithms. A key benefit of the ProSeal laryngeal mask airway (PLMA) is that it has an additional drain tube, which helps prevent aspiration in patients with full stomachs. Trauma patients with cervical spine injuries require immobilization using semi-rigid collars or manual in-line stabilization (MILS). In otherwise healthy individuals, MILS simulates a difficult airway by restricting head and neck movement, which compromises oropharyngeal axis alignment and reduces mouth opening [7].

This study enrolled 60 patients with simulated cervical spine injuries using MILS and randomized them into Group IT and Group ST for PLMA insertion. Fibre-optic evaluation demonstrated no significant difference in placement quality between the groups (p=0.789), indicating equivalence of the two insertion methods. However, Myatra et al. reported superior PLMA positioning with stylet guidance (86% versus 51%), possibly due to insertion performed in the sniffing position [3].

The stylet group showed a higher first-attempt success rate, although this difference did not reach statistical significance (p=0.09). This may be attributed to the stylet stiffening the cuff tip, thereby preventing folding. Similar results were reported by Myatra et al. [3], who found first-attempt success rates of 95% versus 82% in stylet-guided versus traditional techniques, respectively [8]. Other investigators have favoured bougie-guided insertion techniques, reporting even higher success rates, ranging from 96% to 100% [9-12]. Insertion time was significantly shorter in the stylet group (79.37 versus 96.40 seconds, p=0.003), likely reflecting improved shaping of the PLMA. Previous studies corroborate these findings, noting reduced insertion times when experienced anaesthesiologists use adjuncts such as gum elastic bougies [9,13] or fibre-optic bronchoscopes [14].

Regarding haemodynamics, only heart rate at one minute post-insertion differed significantly between groups (p=0.009), with other parameters showing no significant differences. Prior studies employing alternative insertion techniques found no substantial haemodynamic variations [3,11].

Complications such as blood-stained PLMA, postoperative sore throat and hypoxia occurred more frequently in the traditional insertion group, although these differences were not statistically significant (p=0.607). Previous literature similarly reports fewer airway complications with stylet use, likely due to reduced trauma [3,8].

This study has several limitations. It was conducted at a single centre with a modest sample size, limiting the generalizability of the findings. The simulated cervical spine immobilization does not fully replicate the conditions of actual cervical spine injury. Patients with anticipated difficult airways and higher body mass indices were excluded.

## Conclusions

This study highlights that both stylet-guided and introducer tool techniques for ProSeal laryngeal mask airway (PLMA) placement are viable options for airway management in adults requiring manual in-line stabilization during general anaesthesia. Notably, the stylet-guided approach for PLMA placement demonstrates acceptable Brimacombe grades (3 and 4) with distinct clinical advantages, such as reduced technical complexity and the potential to improve the efficiency and safety of airway management, particularly in challenging scenarios. The use of blinded, independent assessment in this study strengthens the validity of these findings. Further large-scale investigations and studies in actual trauma populations are warranted to validate these findings and expand their applicability.
